# Insulin-like growth factor binding protein 5: Diverse roles in cancer

**DOI:** 10.3389/fonc.2022.1052457

**Published:** 2022-11-17

**Authors:** Jennifer A. Waters, Ixchel Urbano, Mikella Robinson, Carrie D. House

**Affiliations:** ^1^ Biology Department, San Diego State University, San Diego, CA, United States; ^2^ Moore’s Cancer Center, University of California, San Diego, San Diego, CA, United States

**Keywords:** cancer, insulin-like growth factor binding protein 5 (IGFBP5), tumor microenvironment (TME), insulin-like growth factor (IGF), extracellular matrix (ECM), therapy resistance

## Abstract

Insulin-like growth factor binding proteins (IGFBPs) and the associated signaling components in the insulin-like growth factor (IGF) pathway regulate cell differentiation, proliferation, apoptosis, and adhesion. Of the IGFBPs, insulin-like growth factor binding protein 5 (IGFBP5) is the most evolutionarily conserved with a dynamic range of IGF-dependent and -independent functions, and studies on the actions of IGFBP5 in cancer have been somewhat paradoxical. In cancer, the IGFBPs respond to external stimuli to modulate disease progression and therapeutic responsiveness in a context specific manner. This review discusses the different roles of IGF signaling and IGFBP5 in disease with an emphasis on discoveries within the last twenty years, which underscore a need to clarify the IGF-independent actions of IGFBP5, the impact of its subcellular localization, the differential activities of each of the subdomains, and the response to elements of the tumor microenvironment (TME). Additionally, recent advances addressing the role of IGFBP5 in resistance to cancer therapeutics will be discussed. A better understanding of the contexts in which IGFBP5 functions will facilitate the discovery of new mechanisms of cancer progression that may lead to novel therapeutic opportunities

## 1 Introduction

The insulin-like growth factor binding proteins (IGFBPs) are a family of secreted proteins that regulate the activity of the insulin-like growth factor (IGF) signaling axis, a critical pathway in growth and development (1). While the entire family of IGFBPs is highly conserved, IGFBP5 is the most conserved across species (2) and plays an important role in development and biological processes of the ovaries, kidneys, mammary gland, bone, and muscle (3–7). Despite the high degree of homology between IGFBPs, each isoform is known to have discrete functions that differentiate it from the other family members. In some tissues multiple IGFBPs are locally expressed and act in disparate ways, such as in bone cells where IGFBP4 and IGFBP5 exhibit opposite effects on proliferation in response to stimulus with IGF (8).

There are six high-affinity isoforms (IGFBP1-6) in the classically defined IGFBP protein family that range in size from 240 to 325 amino acids and share the same basic structure (9, 10). They contain a cysteine-rich C-terminal domain (C), a less conserved linker domain (L), and a cysteine rich N-terminal domain (N), with IGF binding sites within the N and C domains. A closely related protein, IGFBP7, exhibits significantly reduced IGF affinity due to the absence of the conserved cysteine residues present in the C-terminus of IGFBP1-6 (10), but retains IGF-binding activity in the N-terminus. Additionally, several members of the Cyr61 (cysteine-rich protein 61), CTGF (connective tissue growth factor) and NOV (nephroblastoma overexpressed gene), or CCN family, contain IGF binding domains and have been alternatively classified as IGFBP8-10, however these IGFBPs share less structural homology with the IGFBP1-7 isoforms (11).

Studies of IGFBP5 in cancer have resulted in divergent outcomes, where some results suggest it is pro-tumorigenic, and others indicate it is anti-tumorigenic. Evidence suggests IGFBP5 plays a prominent role in the advancement of epithelial cancers including, but not limited to, ovarian, breast endometrioid, prostate, pancreatic, and gastric cancers as well as melanoma and glioblastoma (12). Aberrant expression of IGFBPs in different types of cells results in activation of processes critical to tumorigenesis and metastasis including angiogenesis, migration, and fibrosis; thus, the complex role of IGFBPs in IGF signaling and cancer warrants further discussion. This review will summarize the IGF system and its regulation by IGFBPs, discuss the unique role of IGFBP5 in diseases that share important characteristics with cancer, and expand on what is known about IGFBP5 in cancer progression. Lastly, new clinical discoveries implicating IGFBP5 in therapy resistance will be discussed, with an emphasis on epithelial cancers.

## 2 Regulation of IGF signaling by IGFBPs

### 2.1 Canonical IGF signaling pathway

The canonical function of the IGFBP family members is to bind and regulate bioavailability of IGF. In humans IGF exists in two isoforms, IGF-1 and IGF-2 (13), which act by binding the IGF tyrosine kinase receptor, IGF-1R, and the IGF-2R which has no tyrosine kinase activity (14). Most of the signaling initiated by IGF-1 and IGF-2 is transduced through the IGF-1R, while the IGF-2R is implicated as a scavenger of excess IGF-2 in the extracellular environment (15). The IGFs are also able to bind to the insulin receptor (IR), but do so with less affinity and are primarily strong activators of the IGF signaling axis (16). The signaling pathways activated by the IGF-1R are important for cellular metabolism and growth as well as determination of cell fate.

The majority of IGF1-1 is secreted by the liver, acting as an endocrine hormone, and additional IGF-1 is secreted as a paracrine hormone in most tissues (17). In physiology, IGF-1 acts as a growth hormone, with diffuse roles in diverse cell types. It enhances pancreatic B-cell function; stimulates thyroid hormone production; supports granulosa cell, adrenal cell, and Leydig cell development; and assists M2-like macrophage activation (18). In cancer, extracellular IGF-1 stimulates cancer cell proliferation, survival, cell migration, stem cell-like features, and epithelial to mesenchymal transition (EMT). For example, IGF-1 activates the protein kinase B (AKT) signaling pathway to suppress apoptosis by inhibiting IL1-beta-converting enzyme-like proteases, glycogen synthase kinase 3 (GSK3), and mammalian target of rapamycin (mTOR) activity, all members of a known pathway that regulates apoptotic signaling in cancerous cells (19).

**Figure 1:**
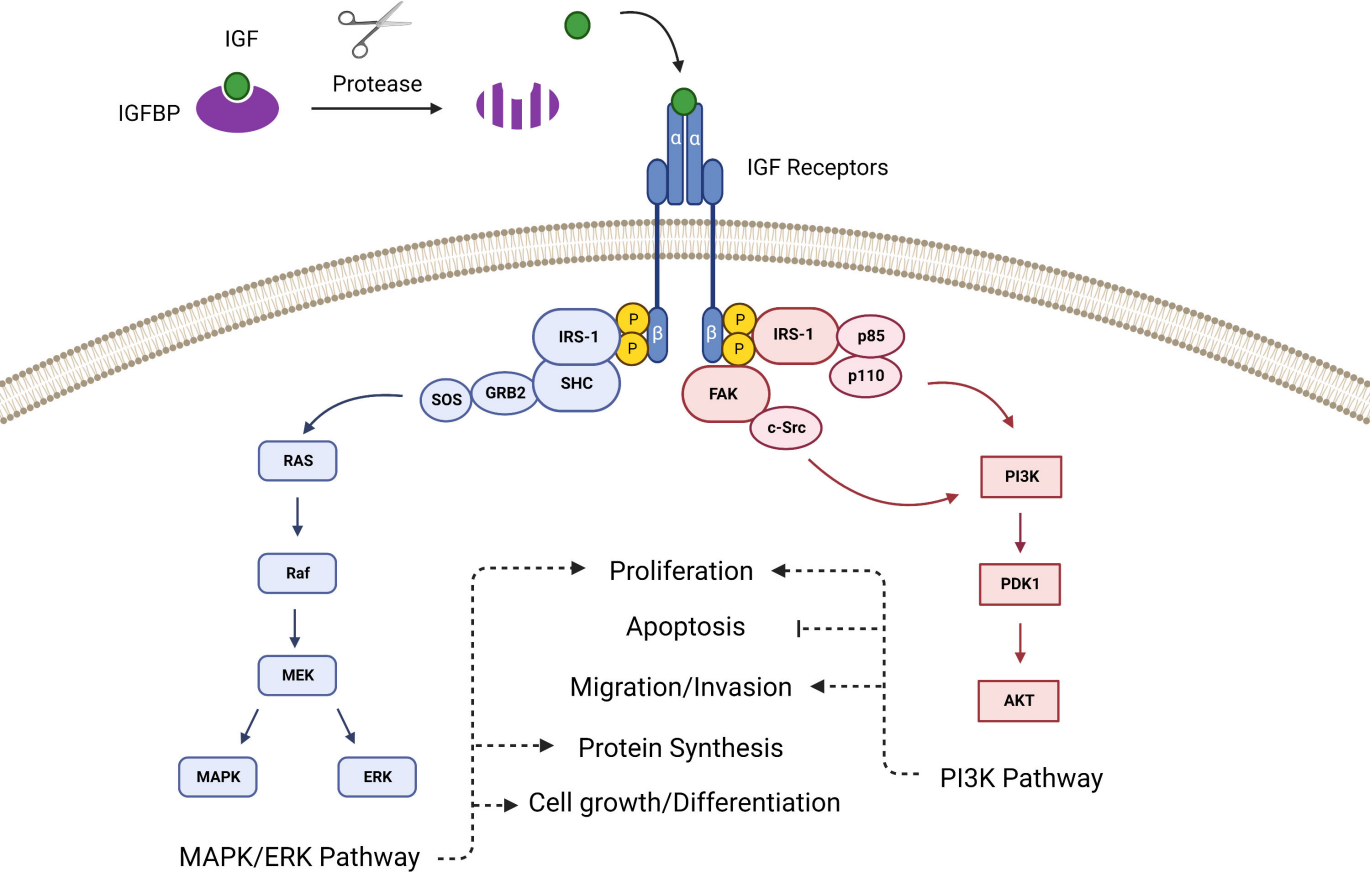
The canonical IGF signaling pathway is activated after proteases cleave the IGFBPs to release IGF, which then binds with the IGF receptor to activate pathways that control proliferation, survival and apoptosis, angiogenesis, differentiation, and development.

The intracellular signaling of the IGF pathway has two primary branches. When the IGF ligand binds the extracellular α-subunits of the mature dimerized IGF-1R, it prompts tyrosine kinases located within the cytoplasmic β-subunits to phosphorylate insulin receptor substrate 1 (IRS-1). IRS-1 contains a recognition motif for src homology 2 domain (SH2) and can therefore bind to proteins containing SH2 domains such as the p85 subunit of phosphatidylinositol-3 kinase (PI3K) and growth factor receptor-bound protein 2 (Grb2) (20, 21). Grb2 links IRS-1 to the Ras pathway *via* Son of Sevenless (SOS), and PI3K links IRS-1 to the AKT pathway, thus IGF ligand binding to the receptor prompts activation of the Ras-MAPK and PI3K-AKT pathways (22) (**Figure 1**). Additionally, the IGF-1R can activate focal adhesion kinase (FAK), which controls cell migration and epithelial to mesenchymal transition in cancer, *via* Proto-oncogene tyrosine-protein kinase Src (c-Src) (23).

### 2.2 IGFBPs and regulation of IGF signaling

The major functional role of IGFBPs is to regulate serum concentrations of IGF, which has a longer half-life when bound by an IGFBP. This interaction thus ensures a reservoir of IGF is available to respond to the changing needs of various tissues; however by sequestering free IGF the IGFBPs negatively regulate IGF signaling (24). When found in serum, IGF is typically part of a ternary complex between IGF, IGFBP3, and the acid-labile subunit (ALS) which together make a carrier protein complex too large to cross the vascular endothelium (25). Although IGFBP3 is the predominant carrier found in this complex, IGFBP5 can also serve in this capacity. The lower molecular weight IGFBP family members (-1, -2, -4, and -6) are found in plasma in binary complexes with IGF that are ~50 kDa and can thus move freely across the vascular endothelium.

IGFBP3 and IGFBP5 serve to fine tune the activation of IGF pathways by maintaining bound IGF in circulation. In response to growth hormone (GH), activating proteases cleave the linker domain of the IGFBPs, thus freeing bound IGF for support of cellular metabolism and growth (26). Thrombin cleavage of IGFBP5 under physiological concentrations produces protein fragments that range in size from 20-24 kDa with no known function and are presumably recycled (27). Matrix metalloproteinase 7 (MMP-7), which is produced in excess by cancer cells, cleaves IGFBP1, -2, -4, -5, and -6 and results in IGF signaling that supports cancer cell growth and survival (28). Pregnancy-associated plasma protein A (PAPP-A), a metalloproteinase that regulates IGF bioavailability through cleavage of IGFBP2, -4, and -5, also has a role in adipose tissue remodeling through its interactions with IGFBP5 (29). Prostate specific antigen (PSA) degrades IGFBP5 to increase IGF bioavailability, the predominant growth factor in the bone microenvironment, which may potentiate pancreatic cancer cell survival and metastasis, although to date this mechanistic work has been restricted to 3T3 fibroblasts and not human cancer cells (30).

## 3 IGFBP5 modulates matrix protein interactions

**Figure 2:**
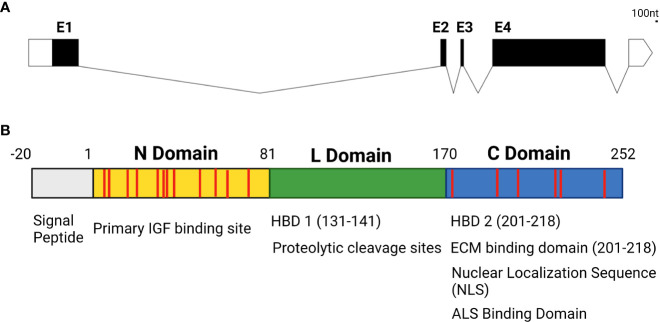
**(A)** Genetic structure of IGFBP5 with untranslated regions denoted in white, exons in black (E1-4) and introns displayed as lines connecting exons. **(B)** Protein structure of IGFBP5 with cysteine residues displayed in red.

While the IGFBP family of proteins is highly conserved and family members have a high degree of structural homology to one another in vertebrates, they do have isoform-specific functional motifs that are exclusive to individual family members. IGFBP5 is arguably the most diverse isoform in terms of its features and functions, with a wide array of IGF-dependent and -independent actions. In addition to IGF binding domains created by disulfide bonds between the cysteines in the N and C domains and binding sites for ALS to maintain IGF concentrations in serum, IGFBP5 contains a nuclear localization sequence (NLS) that permits nuclear translocation, functional domains that enable IGFBP5 to bind to the cell surface, and heparin binding domains (HBD) that enable interactions with the extracellular matrix (ECM) (31–33) (**Figure 2**).

### 3.1 Matrix adhesion

**Figure 3:**
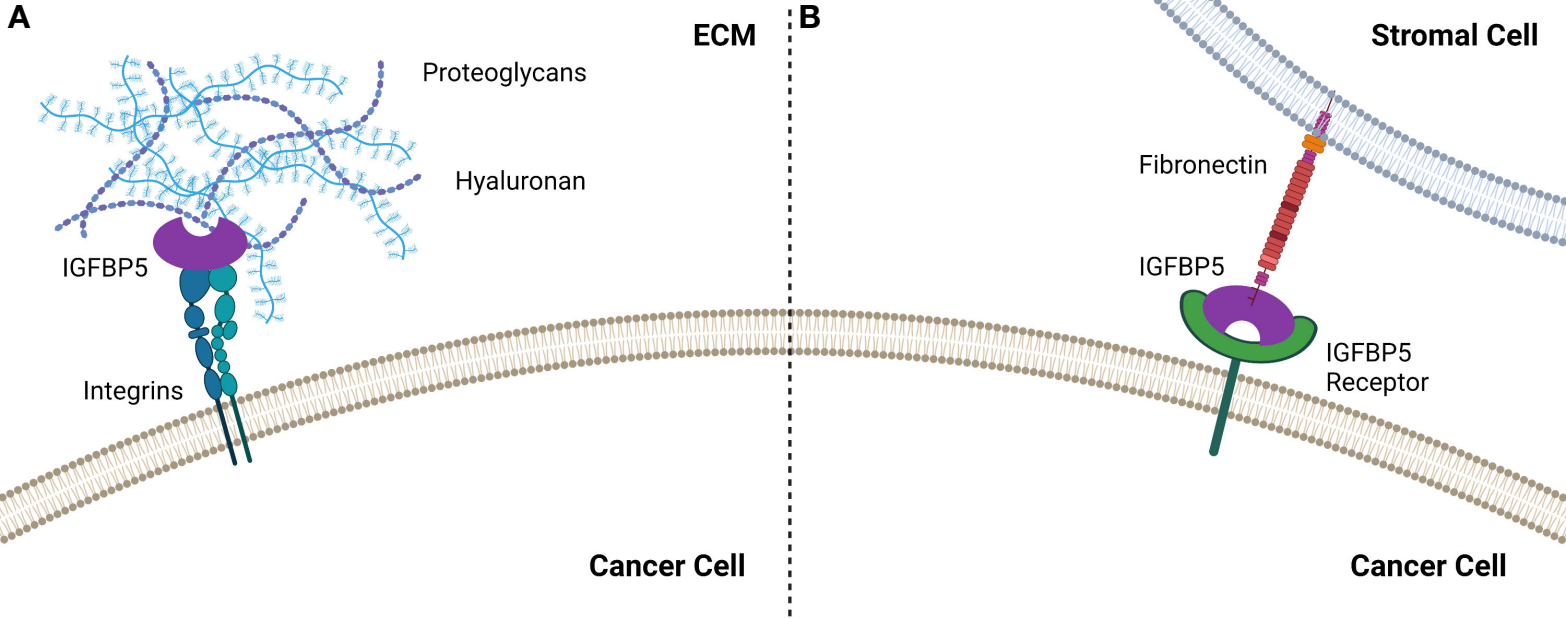
IGFBP5 interacts with the extracellular environment to enable cell attachment by **(A)** binding to integrins which enable attachment via the HBD or **(B)** by interacting with fibronectin the TME, most likely via an unidentified IGFBP5 receptor.

IGFBP5 contains two HBDs, one each in the L and C domains, and the HBD in the C domain enables binding to heparin in the ECM, which has been shown to potentiate growth of fibroblast cells (34). The HBD in the L domain can only interact with heparin when the protein is truncated, specifically when the C domain has been proteolytically cleaved (35). Interestingly, the C-terminal HBD of IGFBP5 stimulates mesangial cell migration (36), suggesting that this region of IGFBP5 promotes cell motility. IGFBP5 also promotes matrix adhesion by α2β1 integrins interacting with the HBD in the C domain in an IGF-independent manner (37) (**Figure 3**).

Increased substrate adhesion by cells in response to IGFBP5 is specific to cellular phenotype, with IGFBP5-induced adhesion elevated in epithelial cells but decreased in mesenchymal cells. Specifically, exogenous treatment with IGFBP5 increased adhesion of epithelial NMuMG cells to laminin, collagen, and fibronectin *in vitro*, while mesenchymal clones derived from the same cell line and treated with IGFBP5 have decreased adhesion to these substrates relative to control (38).

The ability of the HBD to bind the ECM can be modulated by proteolytic cleavage, according to a study using biosensor real-time analysis combined with heparin ligand blotting which concluded that when the protein is intact the C terminal HBD prevents the central HBD from interacting with the ECM (35). This adds to the accumulating evidence suggesting that the truncated C terminal fragment may retain some bioactivity after cleavage and implies that the central HBD does not become active until the HBD located on the C terminal end of the protein has been cleaved, which could have interesting implications *in vivo*.

### 3.2 Cell migration

Beyond its roles in adhesion, the HBD of IGFBP5 has been investigated in mediating cell migration after attachment. The HBD potentiates IGF-1 induced vascular smooth muscle cell (VSMC) migration by interacting with heparin sulfate proteoglycans on the cell surface, and mutations in the HBD cause a reduction in VSMC migration. IGFBP2 and IGFBP4 were unable to induce the same response, indicating that the ability to induce cell migration may be specific to IGFBP5, although this cannot be definitively determined without testing IGFBP3, which also contains an HBD (39). Renal mesangial cells also exhibit increased migration in response to IGFBP5 that is dependent on the HBD and activation of cdc42 and laminin 421, which interact with α6β1-integrin, a process that is impaired by high levels of glucose (36, 40).

IGFBP5 has also been shown to act as an antagonist to transforming growth factor beta (TGFβ), which induces cell migration and wound closure. In NMuMG cells, TGFβ supports epithelial cell adhesion and maintenance of epithelial cell boundaries, while decreased TGFβ induces mesenchymal cell migration (38). Epithelial clones derived from NMuMG cells have decreased wound closure in response to TGFβ, while IGFBP5 increases wound closure both independently and in addition to TGFβ. Mesenchymal clones exhibit increased wound closure in response to TGFβ and while IFGBP5 has no significant impact on its own, it does prevent the increased wound closure caused by TGFβ (38).

### 3.3 Cell surface adhesion

In addition to binding to the ECM, IGFBP3 and IGFBP5 contain functional motifs that enable them to bind to the cell surface (33). Studies using surface plasma resonance (SPR) revealed that both IGFBP3 and -5 interact with the cell binding domain (CBD) of fibronectin (41), which is a preferred substrate for epithelial cells treated with IGFBP5.

## 4 IGFBP5 in development and disease

**Figure 4:**
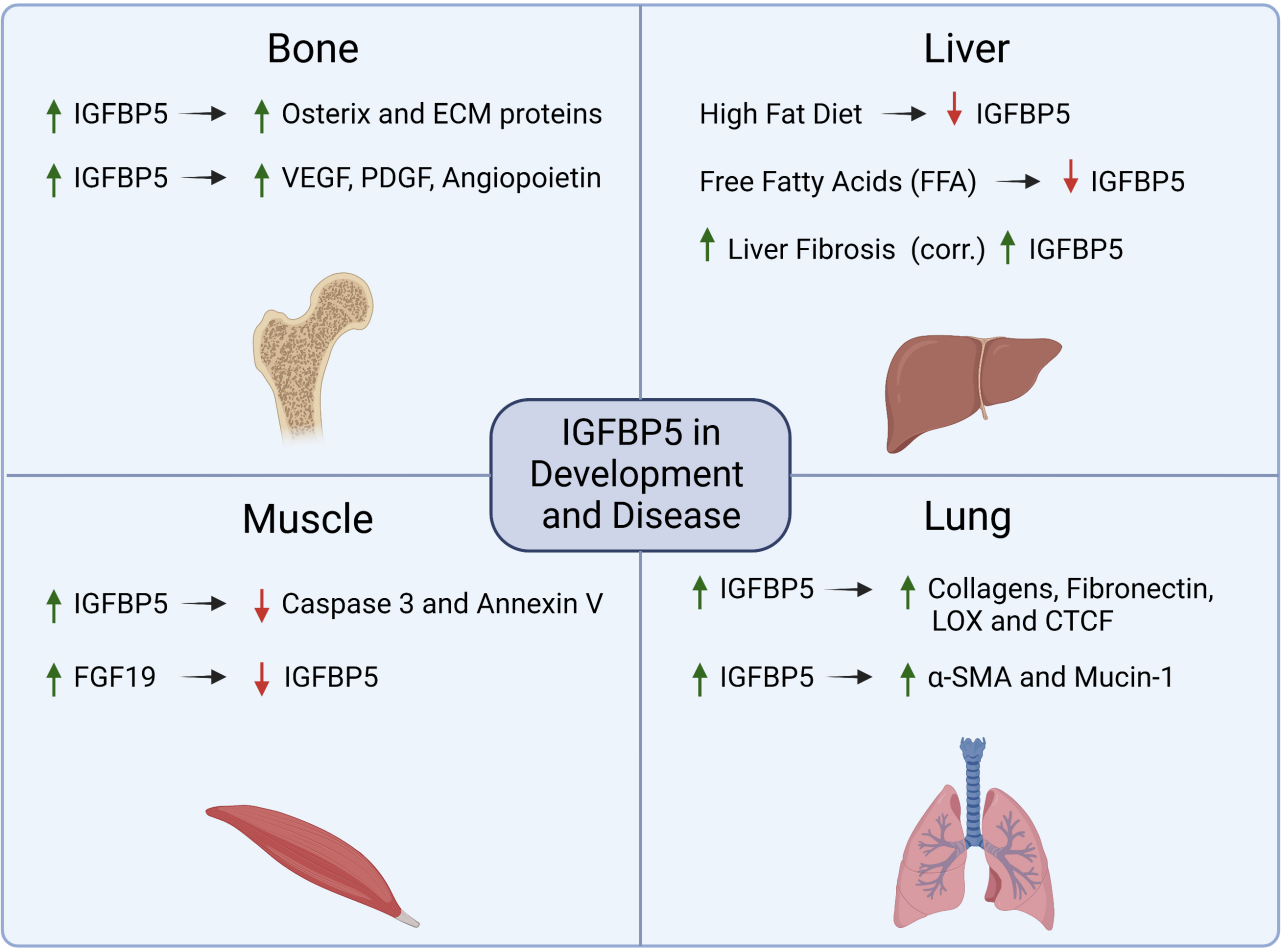
Summary of mechanisms that are regulated by IGFBP5 or which regulate IGFBP5 in physiology and non-cancer diseases.

IGFBP5 is the most conserved IGFBP family member with 97% homology between the murine and human isoforms (42). Several of the hallmarks of cancer, including sustained cell proliferation and resistance to apoptosis, result from dysregulation of genes, such as IGFBP5, that are key in developmental pathways and can thus facilitate aberrant hyperplasia (**Figure 4**). In this section, we will review the role of IGFBP5 in developmental biology as well as its role in non-cancer diseases to highlight some of the functional characteristics of IGFBP5 that make it a prime candidate for investigation in cancer. An extended review of IGFBP5 in physiology and endocrinology has recently been published and will not be covered here (43).

### 4.1 IGFBP5 regulates developmental pathways in bone and muscle

In human development, IGFBP5 acts in an IGF-dependent manner to promote osteogenesis and myogenesis. During osteogenesis, IGFBP5 is upregulated in mesenchymal stem cells (MSCs) that reside in craniofacial tissues to enhance differentiation and periodontal tissue regeneration. Upon osteogenic induction, lysine (K)-specific demethylase 6B (KDM6B) demethylates histone K27 on the promoter of IGFBP5 leading to transcription of IGFBP5, expression of matrix proteins, and induction of osteogenic transcription factors such as osterix (44). IGFBP5 also binds to an uncharacterized cell surface receptor in osteoblasts (45) to stimulate mitogenesis *via* an IGF-independent mechanism. In dental pulp cells, an osteogenic stem cell lineage derived from MSCs, IGFBP5 promotes osteogenic differentiation by enhancing angiogenesis and neurogenesis *via* increased expression of VEGF, PDGF, and angiopoietin (46).

Overexpression of wild-type IGFBP5 or a mutant non-IGF binding IGFBP5 in murine myoblasts revealed that differentiation into multinucleated myotubes required IGFBP5 to bind to IGF-1. The same study investigated whether the mutation impacted the effect of IGFBP5 overexpression on proliferation and apoptosis, and while IGFBP5 does not appear to individually regulate cell proliferation, it does have anti-apoptotic activity that is not dependent on IGF. This anti-apoptotic phenotype was discovered in differentiating myotubes by evaluating both caspase 3 activity and annexin V staining, both of which were decreased in IGFBP5 overexpressing cells (47).

Cerebral palsy (CP) is a perinatal disease that causes developmental defects in skeletal muscle and decreased mobility, and clinical samples of wrist muscle in children with CP have elevated gene expression of IGFBP5. In a preclinical model for CP, rats with decreased body growth, defects in mobility patterns and speed, muscle atrophy, and elevated IGFBP5 responded to treatment with fibroblast growth factor 19 (FGF19), which rescued the increase in IGFBP5 expression in CP rats as well as their functional defects (48). This suggests that IGFBP5 could be a target in treatment of neurodegenerative and musculoskeletal disease.

### 4.2 IGFBP5 is implicated in diseases with increased fibrosis and adipogenesis

#### 4.2.1 Idiopathic pulmonary fibrosis

Beyond its role in development, IGFBP5 has been investigated in pathologies such as idiopathic pulmonary fibrosis (IPF) (49), liver disease (50), obesity (51), and the negative impacts of aging (52). Pulmonary fibrosis is characterized by persistent overproduction of ECM components which causes decreased pulmonary function due to increased tissue stiffness. Activated pulmonary fibroblasts overexpress IGFBP5 which induces expression of ECM proteins such as collagens, fibronectin, connective tissue growth factor (CTGF), and lysyl oxidase (LOX) (53). Mice that were administered an adenoviral vector to overexpress IGFBP5 have increased collagen deposition in their lung tissue, which was not observed in mice treated with adenoviral particles for IGFBP3 or control particles lacking cDNA, indicating that this phenotype is IGFBP5 specific (54).

In addition to increased fibrosis, the same study showed that overexpression of IGFBP5 led to mononuclear cell recruitment and an increase in epithelial to mesenchymal transition (EMT) by alveolar epithelial cells, resulting in increased Mucin-1 and alpha-smooth muscle actin (α-SMA) expression. In a transgenic mouse model with human IGFBP5 (hIGFBP5) CRISPR-Cas9 knock-in, mice expressing hIGFBP5 had increased expression of collagen 3A1, fibronectin and LOX. There was also a positive correlation between hIGFBP5 copy number and increases in dermal thickness and deposition of ECM proteins observed in lung tissue (55). Taken together these results indicate that IGFBP5 regulates ECM deposition in pulmonary disease.

#### 4.2.2 Non-alcoholic steatohepatitis

The increase in fibrosis observed in lung diseases such as IPF is also a hallmark of liver disease. Non-alcoholic fatty liver disease (NAFLD) is quickly becoming one of the most prevalent forms of liver disease as obesity becomes more widespread (56), and is characterized by elevated fat deposition and increased inflammation and fibrosis in the liver (56). The concentration of IGFBP5 in serum from NAFLD patients is positively correlated with fibrosis and steatosis in the liver and has been proposed to be a marker for progression from NAFLD to a more serious liver disease, non-alcoholic steatohepatitis (NASH) (57). However, *in vitro* studies of fatty liver disease using cultured hepatocytes fed with free fatty acids and *in vivo* studies maintaining mice on a high fat diet show that IGFBP5 is decreased in NAFLD models, and overexpression of IGFBP5 resulted in reduced expression of proteins associated with lipogenesis (50).

The correlation between serum levels of IGFBP5 and disease phenotypes that indicate progression of NAFLD to NASH is informative and could potentially point to a biomarker of disease progression, but it should be noted that the serum concentration of IGFBP5 does not reflect liver-tissue specific IGFBP5 expression. This provides a clear example of the context dependence of IGFBP5, with one study suggesting that increased IGFBP5 in serum is correlated with NAFLD progression and severity, while another suggests the opposite based on expression in diseased liver tissue. While further work is needed to elucidate the role(s) of IGFBP5 in the progression of NAFLD and NASH, it does appear that expression of IGFBP5 impacts NAFLD patient prognosis and regulates hepatocellular lipogenesis.

In addition to regulating lipogenesis in the liver, IGFBP5 has been linked to the reemergence of adipocytes in murine breast tissue after weaning. In the absence of IGFBP5, mice experience delayed mammary gland involution, normally characterized by loss of mammary epithelial cells, degradation of basement membranes and subsequent repopulation of adipocytes. Tunel staining of the glandular epithelial cells in IGFBP5 null mice revealed a decrease in apoptotic activity leading to delayed involution (58). IGFBP5 null mice also exhibit an increase in body size and glucose intolerance, which is exacerbated when mice are maintained on a high fat diet (51).

The implication of IGFBP5 in these pathologies result from its activity in regulating cell growth and differentiation, ECM secretion, and immune cell infiltration. These findings suggest that IGFBP5 can stimulate stromal cell fibrosis and induce chemotaxis of immune cells, both of which are key players in the tumor microenvironment (TME) in cancer. These and other functional roles which implicate IGFBP5 in the progression of different cancers will now be discussed.

## 5 Expression patterns of IGFBP5 in cancer

### 5.1 Cancer cell lines

**Figure 5:**
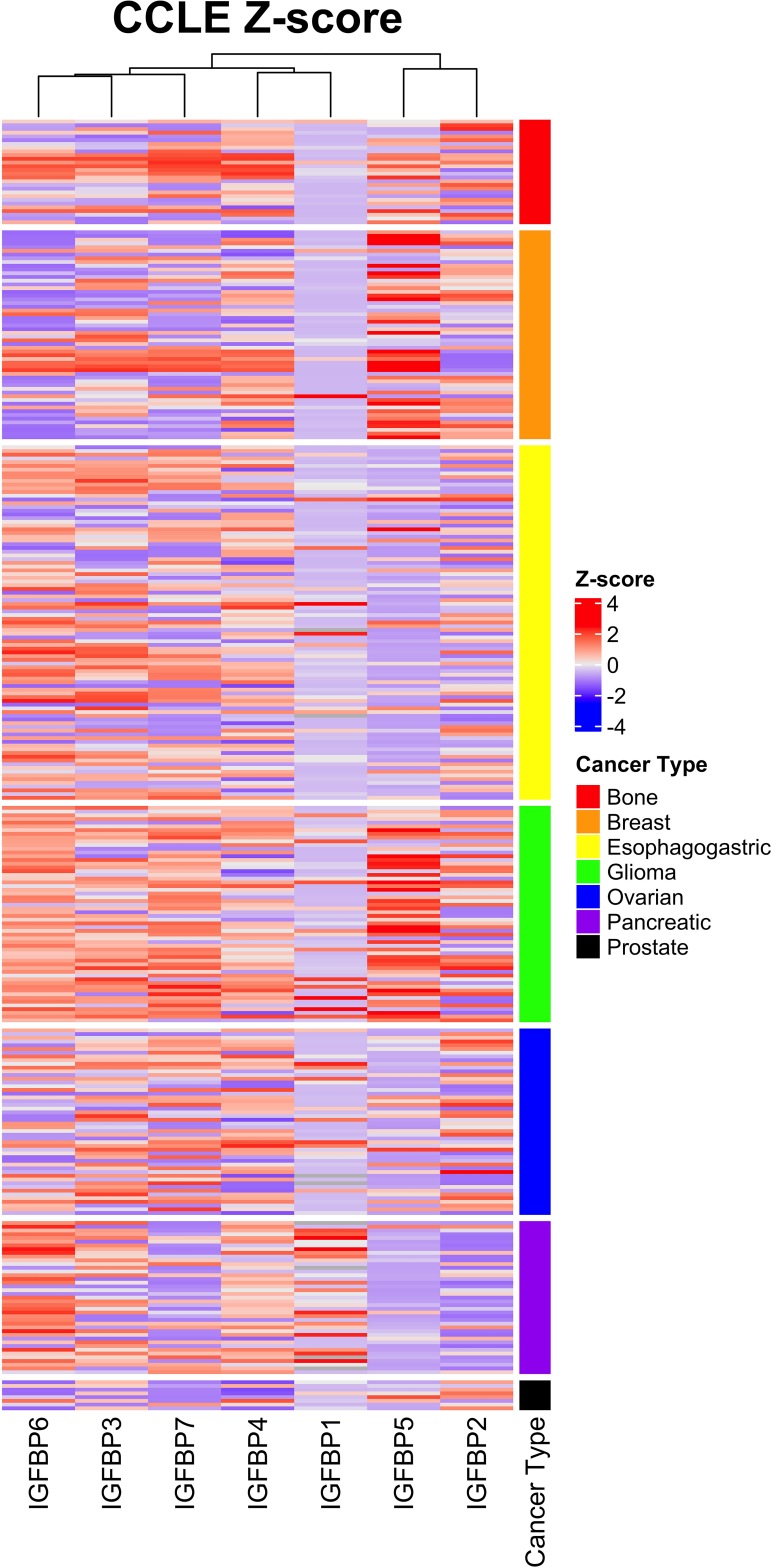
Expression of IGFBP5 in the Cancer Cell Line Encylopedia (CCLE) displayed as z-scores and clustered by cancer subtype. Normalized gene expression (mRNA expression z-scores relative to all samples, log RNA Seq RPKM) values and cancer subtype were downloaded from The Cancer Genome Atlas on cbioportal and reconstructed in excel by unique patient ID. Heatmap was generated using ComplexHeatmap in rstudio. The cancer subtype cluster are shown above with the indicated colors.

Expression of IGFBP5 and the other IGFBPs in normal and cancerous tissues and cells is varied and regulated by a variety of factors including transcriptional regulators, chromatin modifiers, non-coding RNAs (59). To assess the complexity of IGFBP expression in cancer-derived cell lines, we analyzed the previously published Cancer Cell Line Encyclopedia (CCLE) *via* cBioportal (60, 61). This CCLE heatmap shows a complexity of IGFBP expression in the cancer subtypes (**Figure 5**). While some trends are more visually apparent, such as the enrichment within glioma and breast cancer cell lines for high-IGFBP5 expressing cell lines, expression within other types of cancer such as bone is more nuanced and contains both high- and low-expressing cell lines. IGFBP5 expression was consistently lower in cell lines derived from ovarian, pancreatic and esophagogastric cancers, which is consistent with reports from a study of patient samples which reported low levels of IGFBP5 expression in cancer tissue relative to normal ovarian surface epithelium (62).

### 5.2 Clinical samples

**Figure 6:**
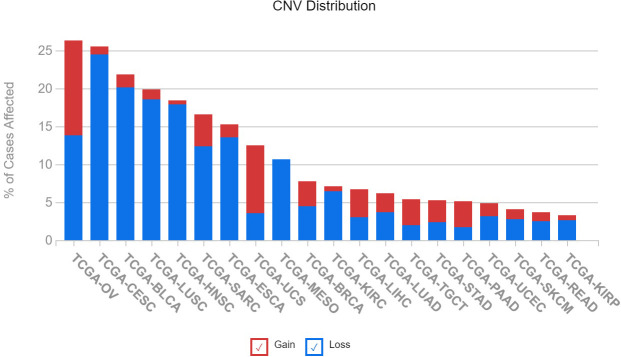
Copy number variation of IGFBP5 is most prevalent in gynecologic cancers in patients from The Cancer Genome Atlas (TCGA) and can result in both gain and loss of function. Illustrates the number of cases of altered IGFBP5 gene across 20 TCGA cancer types (BLCA, bladder urothelial carcinoma; BRCA, breast invasive carcinoma; CESC; cervical squamous cell carcinoma and endocervical adenocarcinoma; ESCA, esophageal carcinoma; HNSC, head and neck squamous cell carcinoma; KIRC, kidney renal clear cell carcinoma; KIRP, kidney renal papillary cell carcinoma; LIHC, liver hepatocellular carcinoma; LUAD, lung adenocarcinoma; LUSC, lung squamous cell carcinoma; MESO, mesothelioma; OV, ovarian serous cystadenocarcinoma; READ, Rectum adenocarcinoma; SARC, sarcoma; SKCM, skin cutaneous melanoma; STAD, stomach adenocarcinoma; TGCT, testicular germ cell tumors; PAAD, pancreatic adenocarcinoma; UCEC, uterine corpus endometrial carcinoma; UCS, uterine carcinosarcoma).

The varying expression levels of IGFBP5 observed in cancer cells lines is also observed in patient samples in previously published datasets. We queried 20 different cancer subtypes for the number of patients with copy number variations in The Cancer Genome Atlas (TCGA). An assessment of IGFBP5 revealed that the greatest rate of CNVs is found in ovarian cancer, with over 25% of patients possessing either an increase or decrease in copy number of the IGFBP5 gene. It is interesting to note that although ovarian cancer has the highest rate of CNVs, there is no specific trend for copy number gain or loss (**Figure 6**). In the other cancers with the highest rates of affected cases, which include cervical squamous cell carcinoma, bladder urothelial carcinoma, lung squamous cell carcinoma, and head and neck squamous cell carcinoma, there is a higher rate of loss compared to gain of copy number in the variants detected.

In ovarian cancer, a study investigating mRNA and protein levels of IGFBP family members in normal and cancerous tissues found that IGFBP5 was decreased in cancerous tissue relative to normal ovarian tissue, however the same study reported a strong association between increased expression of IGFBP5 and decreased progression-free survival and overall survival in all subtypes of ovarian cancer (62). Further analysis revealed the prognostic value of IGFBP5 in ovarian cancer was more significant in patients receiving paclitaxel or paclitaxel and carboplatin combination therapy than carboplatin as a monotherapy, indicating that IGFBP5 may play a role in therapeutic responses to microtubule-stabilizing agents used to treat ovarian cancer (62).

A tissue microarray analysis of breast carcinomas revealed cytoplasmic staining for IGFBP5 was elevated in samples from patients with invasive carcinomas relative to normal breast epithelium or benign breast tissue (63). Increased staining intensity was more prevalent in patients with nodal metastasis (T1N1) versus those with negative nodes (T1N0), and was correlated with expression of the progesterone receptor (PR) and human epidermal growth factor receptor 2 (HER2) (63).

IGFBP5 protein expression is elevated in pancreatic ductal adenocarcinoma (PDAC) tissue and is strongest at the leading edge of the tumor, when compared to IHC staining of non-malignant duct specimens (64). Pathway analysis found that tissues which express elevated IGFBP5 are also enriched for adhesion and ECM gene programs. Specific hub genes that cluster with IGFBP5 and are similarly overexpressed in PDAC tissue include matrix metalloproteinase 14 (MMP14) and collagen 12A1 (COL12A1) (65). Additionally, single-cell sequencing revealed that IGFBP5 was enriched in pancreatic circulating tumors cells (CTCs) compared to bulk tumor. This data suggests the ability of low-expressing bulk tumor cells to modulate IGFBP5 expression as needed during stages of the metastasis process. These CTCs expressing IGFBP5 also had increased expression of stem cell associated genes aldehyde dehydrogenase 1A2 (ALDH1A2) and Kruppel-like factor 4 (Klf4) (66), which could indicate a cancer stem-like cell phenotype, a state thought to modulate the ability of cancer cells to seed new tumors.

### 5.3 Cancer-associated stromal cells

Interestingly, IGFBP5 expressing CTCs were localized at the epithelial-stromal boundary, indicating that the stromal cells surrounding the tumor may be involved in modulating the expression of IGFBP5. Stromal cells including fibroblasts (67) and preadipocytes (68) secrete IGFBP5. Studies in fibroblasts have shown that IGFBP5 can potentiate fibroblast growth by sequestering the IGF ligand in complex within the matrix (34), a process that relies on proteolytic cleavage of the matrix bound complex between IGF and IGFBP5. Proteolysis plays a significant role in regulation of IGF signaling, and proteases such as MMP-2 can cleave IGFBP5, thereby freeing IGF for activation of proliferative and anti-apoptotic signaling pathways in the local milieu (69).

IGFBP5 has also been shown to drive premature cellular senescence in fibroblasts which results in a phenotype similar to cancer-associated fibroblasts (CAFs) (70). Senescence can be induced in human dermal fibroblasts *via* treatment with a Rho-kinase (ROCK) inhibitor which enables cells to resist apoptosis by undergoing growth arrest. After extended exposure to ROCK inhibitors fibroblasts eventually acquire a CAF-like genetic and secretory profile (67). RNA sequencing of senescent fibroblasts revealed upregulation of IGFBP5, and stimulation with exogenous IGFBP5 independently upregulated the senescence marker SA-β-gal. IGFBP5 knockdown blocked the increase in senescence caused by either the ROCK inhibitor or exogenous IGFBP5 (67), demonstrating that IGFBP5 is required for fibroblasts to acquire a senescent or CAF-like phenotype. Additionally, mass spectrometry of cell culture supernatants revealed that patient derived CAFs secrete more IGFBP5 than normal mammary fibroblasts, and the secreted IGFBP5 inhibits anoikis by stabilizing myeloid leukemia 1 (MCL-1) which is upstream of BCL-2 (71).

Taken together, these findings provide evidence that IGFBP5 in normal tissue facilitates differentiation, fibrosis, and adhesion, while inhibiting apoptosis. IGFBP5 acts through numerous signaling pathways, including but not limited to VEGF, NF-kB, AKT, and TGFβ, each of which may induce an independent phenotype in cancer. **Table 1** highlights studies investigating the role of IGFBP5 in cancer published in the last 15 years and classifies each as either anti-tumorigenic, pro-tumorigenic, or undetermined. Subsequent sections will explore these studies in greater depth.

**Table 1:** Evidence for anti-tumorigenic, pro-tumorigenic, or undetermined functions of IGFBP5 in cancer.

Cancer Type	Source of Tissue/Cell Line	Summary of findings regarding the role of IGFBP5 in cancer	Effect on Cancer	Reference	Year
Ovarian	OvCAR3, SKOV3, PA-1, HUVEC cell lines	C-terminus of IGFBP5 exhibits anti-cancer activity through inhibition of angiogenesis by down-regulating VEGF.	Anti-tumorigenic	(72)	2016
	OVCAR5, SKOV3, A2780, OV2008, & HcerEpiC cell lines	circPIP5K1A downregulates miR-661, which then increases expression of IGFBP5 to promote cancer progression.	Pro-tumorigenic	(73)	2019
	SKOV-3 cell line	Tumor growth and vascularity were decreased with IGFBP5 overexpression in a subcutaneous xenograft.	Anti-tumorigenic	(74)	2008
	Patient samples in databases (GEPIA, Oncomine, HPA)	Overexpression of IGFBP5 predicted worsened OS and PFS in SOC and EOC, however PFS for grade III OC was inversely correlated with IGFBP5.	Pro-tumorigenic	(62)	2020
Breast	MCF-7 cell line	Single allelic enCNV of the 2q35 breast cancer risk loci repress expression of IGFBP5, which can be induced by oestrogen.	Anti-tumorigenic	(75)	2016
	Patient samples (11 Luminal A, 11 Luminal B, 12 Her2 and 14 Basal-like)	IGFBP5 mRNA-negative samples have elevated expression of miR-140-5p. miR-140-5p expression is increased in tumor tissue relative to normal tissue.	Anti-tumorigenic	(76)	2015
	MDA-MB-435 cell line	Nuclear IGFBP5 decreases cell growth and motility while cytoplasmic IGFBP5 increases proliferation and migration.	Pro-tumorigenic	(77)	2009
	MCF-7, T-47D, MDA-MB-361, MDA-MB-415, MDA-MB-231, BT-549, Hs 578T, & MDA-MB-157 cell lines	H3K27me3 demethylase KDM6B promotes IGFBP5 expression which leads to epigenetically induced resistance to PI3K inhibiters. Combination therapy targeting KDM6B and PI3K results in cancer cell apoptosis by downregulating IGFBP5.	Pro-tumorigenic	(78)	2018
	MCF-7 cell line	IGFBP5 is released from apoptotic cells and promotes adhesion to the ECM by association with the heparin binding domain *via* an IGF-independent mechanism. IGFBP5 also increases e-cadherin expression and inhibits cell migration.	Anti-tumorigenic	(37)	2012
	Human patient samples (breast cancer vs. normal adjacent tissue)	Higher expression of IGFBP5 is correlated with advanced tumor grades and thus IGFBP5 is considered pro-tumorigenic. Tissues with the highest IGFBP5 expression also coordinately overexpress COL1A1 and MMP11.	Pro-tumorigenic	(79)	2015
	Human patient tissue sample from breast carcinoma	IGFBP5 methylation was not changed between tumor and adjacent normal tissue, however IGFBP5 expression was positively correlated with G2 tumor stage and ER+ patients.	Undetermined	(80)	2016
	MCF-7, Bt474, T47D cell lines	Stromal cells induce IGFBP5 downregulation in ERα-positive breast cancer cells which results in desensitization to anti-estrogen therapies.	Anti-tumorigenic	(81)	2015
Gastric	Gastric cancer cell lines and primary gastric cancer tissues	PKNOX2 positively regulates IGFBP5 expression *in vitro* and *in vivo* by acting as a tumor suppressor.	Anti-tumorigenic	(82)	2019
	GIST-T1 & GIST882 cell lines	DOG1 blockade delays xenograft growth and upregulates IGFBP5 in gastrointestinal stromal tumors (GIST), and imatinib resistant cells downregulate IGFBP5 mRNA.	Anti-tumorigenic	(83)	2013
Pancreatic	Patient samples from PDAC and non-malignant tissue	IGFBP5 is upregulated in pancreatic ductal adenocarcinoma and expression is increased in the islet cells closest to the periphery of the tumor.	Pro-tumorigenic	(64)	2006
	Human peripheral lymphocytes	Observed significant association between pancreatic cancer risk and polymorphisms in IGF1, IGF1R & IGFBP1 but SNPs in IGFBP5 were not significant.	Undetermined	(84)	2012
	BxPC-3 and PANC-1 cell lines	IGFBP5 overexpression accelerated cell cycle progression and activated AKT in BxPC-3 cells but caused cell cycle arrest and reduced AKT signaling in PANC-1 cells.	Undetermined	(85)	2009
	KPC mouse model	Circulating tumor cells (CTCs) are enriched for IGFBP5 and the IGFBP5 expressing CTCs are localized to the epithelial-stromal boundary.	Pro-tumorigenic	(66)	2014
Cervical	Human patient samples	IGFBP5 expression is down-regulated in invasive cervical carcinoma and up-regulated in cervical intraepithelial neoplasia.	Undetermined	(86)	2009
Prostate	PC3, DU145 cell lines	IGFBP5 expression in prostate cancer tissue and prostate carcinoma is significantly lower than normal tissue, while overexpression of IGFBP5 enhances radiosensitivity.	Anti-tumorigenic	(87)	2020
Melanoma	HEMn-LP, A375, A2058 UACC903 cell lines	IGFBP5 suppresses pathogenesis and metastasis of malignant melanoma through inhibition of ERK1/2 and P38-MAPK pathways.	Anti-tumorigenic	(88)	2015
Glioblastoma	U87, U251, LN229 cell lines	IGFBP5 expression increases in advanced stages of GBM. It regulates EMT by inhibiting cell proliferation and promoting cell invasion *via* the AKT signaling pathway.	Pro-tumorigenic	(89)	2020
	Patient derived neurosphere cell lines	Increased expression of IGFBP5, IGFBP3 and IRE1 identifies non-responders that are resistant to apoptosis *via* the UPR.	Pro-tumorigenic	(90)	2021
Colon	LoVo, SW480, HCT116, Caco-2, SW620 cell lines	Anticancer activity of tetrandrine in colon cancer may be mediated by downregulation of IGFBP5 expression that inactivates the canonical Wnt pathway.	Pro-tumorigenic	(91)	2014
Head & Neck	Human squamous cell carcinoma of the head and neck	IGFBP5 -1195T>C polymorphism is functional and could be a biomarker for susceptibility to late-stage squamous cell carcinoma of the head and neck.	Undetermined	(92)	2010
Lung	Non-small cell lung cancer (NSCLC) patient samples	Increased IGFBP2 and IGFBP5 mRNA levels were correlated with increased overall survival in squamous cell carcinoma patients.	Anti-tumorigenic	(93)	2019
Thyroid	TCP-1, BCPAP, HEK293T cell lines	IGFBP5 expression is elevated in papillary thyroid carcinoma. Overexpression of IGFBP5 partially abrogates miR-204-5p-induced effects on tumorigenesis.	Pro-tumorigenic	(94)	2015
Renal	Patient samples	IGFBP5 is downregulated in KIRP kidney tissues and low levels of IGFBP5 are correlated with a longer survival time.	Pro-tumorigenic	(95)	2019
Osteosarcoma	HEK293, MG63, 143B, MNG/HOS TE85 cell lines	IGFBP5 inhibits cell proliferation, migration, and invasion *in vitro* indicating that it is anti-tumorigenic in osteosarcoma.	Anti-tumorigenic	(96)	2011
	HEK293, Human OS 143B cell lines	Over-expression of IGFBP5 inhibits cell proliferation and primary tumor growth and induces apoptosis.	Anti-tumorigenic	(97)	2013
Liver	HCC patient tissue samples	Mybbp1a in complex with DNMT1 induces hyper-methylation of CpG islands of IGFBP5, which reduces IGFBP5 secretion thus activating the IGF/AKT signaling pathway.	Anti-tumorigenic	(98)	2019

## 6 Implications in for IGFBP5-ECM interactions in cancer

Fibrosis is linked to up to 20% of cancers and results in a chronic state of inflammation and increased deposition of matrix proteins (99). A cDNA microarray comparing breast cancer tissues with adjacent normal tissues discovered not only elevated IGFBP5 expression in the malignant tissue, but also correlated increases in COL1A1 and MMP11, indicating that IGFBP5 may play a role in ECM remodeling to create a pro-tumorigenic stromal compartment (79). A practical example of this collaborative relationship between IGFBP5 and the ECM in cancer is shown in a study demonstrating IGFBP5 can bind directly to vitronectin to facilitate adhesion of breast cancer cells to the mesenchymal ECM *via* interaction with α2β1 integrins, as opposed to the αVβ3 integrins that canonically act as the vitronectin receptor (37).

Studies of the ovarian cancer TME have identified vitronectin and fibronectin as key facilitators of cancer cell attachment to the mesothelium that lines peritoneal organs, indicating these mechanisms may be important in peritoneal cancers as well (100). This is interesting given that IGFBP3, which has some compensatory mechanisms in response to loss of IGFBP5, enhances attachment to fibronectin. This compensatory mechanism was discovered when mice with homozygous deletion of the IGFBP5 gene were shown to have minimal developmental defects aside from delayed mammary gland involution, largely due to compensatory increases IGFBP3 expression (58). IGFBP5 also enhanced attachment of breast cancer cells to thrombospondin, and conferred resistance to ceramide induced apoptosis (101). Lastly, cell attachment to laminin or collagen IV by breast cancer cells increased by exogenous addition of either recombinant IGFBP5 or IGFBP4 (101).

## 7 Tumor suppressor activity of IGFBP5

IGFBP5 has been suggested to act as a tumor suppressor in several cancers including ovarian cancer (72), melanoma (88), and osteosarcoma (97). This was first investigated in human umbilical vein endothelial cells (HUVECs), a useful but non-cancerous model for studying angiogenesis and cell migration. IGFBP5 overexpression is antiangiogenic and abrogates VEGF induced increases in proliferation and tube formation by HUVEC cells as well as invasion by HUVECs through Matrigel, which results from decreased phosphorylation of AKT and eNOS (74). The same study demonstrated that intratumoral injection of IGFBP5 into subcutaneous xenografts with SKOV3 ovarian cancer cells prevented further tumor growth and inhibited vascularization as measured with CD31 staining (74), setting a solid framework for future studies investigating the role of IGFBP5 as a potential tumor suppressor.

When investigated in endometrioid cancer cells, specific regions of the protein were more potent tumor suppressors than others. Endometrioid ovarian cancer cells expressing a truncated peptide for each domain of IGFBP5 (C, L and N) had varying effects on tumor growth in a subcutaneous injection model. Tumor growth was attenuated in cells expressing the c-terminal peptide, decreased in cells expressing the n-terminal peptide, and rapidly increased in cells expressing the linker peptide. Treating patient derived xenografts with the c-terminal peptide decreased tumor growth and expression of CD31 indicating that the peptide reduced vascularization. Mechanistically, the c-terminal peptide inhibited expression of VEGF, interleukin-6 (IL-6) and tumor necrosis factor alpha (TNF-α), suggesting the antiangiogenic phenotype resulted from reduced VEGF and NF-kB signaling (72). Although the differential activity of the three regions of IGFBP5 is compelling, it is not clear whether this truncated c-terminal product of IGFBP5 exists in clinical samples.

IGFBP5 additionally suppressed tumor growth and metastasis in several models of osteosarcoma and melanoma (96, 97). IGFBP5 has been identified as a hub gene in osteosarcoma along with origin recognition complex subunit 6 (ORC6), minichromosome maintenance 10 replication initiation factor (MCM10), MET proto-oncogene, receptor tyrosine kinase (MET) and centromere protein F (CENPF) (102). However, IGFBP5 had the least significant prognostic value of the hub genes identified. Despite the lack of prognostic significance, IGFBP5 was shown to play an important role in osteosarcoma using several *in vivo* models. IGFBP5 overexpression in an orthotopic xenograft inhibited tumor growth and pulmonary metastasis while siRNA mediated silencing enhanced pulmonary metastasis after cell injection into the tibia (96). Overexpression of IGFBP5 in A375, a malignant melanoma cell line, dramatically reduced tumor size in a subcutaneous xenograft model and reduced tumor growth and metastasis in a pulmonary metastasis model (88). The same study found that IGFBP5 overexpression blocked migration, invasion, and colony formation *in vitro*. Interestingly, clinical data shows that IGFBP5 is overexpressed in melanoma compared to normal pigmental nervus (88).

In breast cancer, Wnt mediates the transition to therapy-resistance by maintaining features of stemness and, in breast cancer cells, negatively regulates IGFBP5 (103). Wnt is a highly conserved signaling pathway that regulates stem cell populations and cell fate determination during development, and like IGFBP5, plays a role in mammary gland involution. Wnt activated tumors exhibit aberrant IGF signaling that promotes proliferation and tumor growth, while inhibition of Wnt signaling with a soluble frizzled8 fusion protein that competes for Wnt1 binding caused activation of IGFBP5, downregulation of IGF signaling, and tumor regression (103). When IGFBP5 knockdown *via* shRNA is combined with Wnt1 inhibition using the frizzled8 fusion protein, the effect of the fusion protein is diminished and tumor volumes increase over time, indicating that the mechanism by which Wnt1 promotes tumor growth is dependent on downregulation of IGFBP5 in a breast cancer model (103).

## 8 Oncogenic activity of IGFBP5

In addition to the large body of evidence that indicates IGFBP5 acts as a tumor suppressor, there is considerable evidence that it has oncogenic roles. In ovarian cancer, as previously discussed, increased IGFBP5 was positively correlated with decreased overall survival, suggesting it is important for cancer progression (62). In breast cancer, IGFBP5 is oncogenic specifically when it is localized within the nucleus, a phenomenon observed primarily in cell lines engineered to ectopically overexpress IGFBP5 (77). Generation of cell lines that trap IGFBP5 in the nucleus by mutating the NLS resulted in increased growth and migration, indicating that subcellular localization of IGFBP5 is important in determining its role in cancer (77). However, since IGFBP5 is localized primarily to the cytosol in patient tissues, it remains unclear whether nuclear localization of IGFBP5 contributes to breast cancer progression (77). A review that specifically explores the role of IGFBP5 in breast cancer details some of the studies that led to this work in greater depth (59).

Wnt signaling, like the cases previously discussed regarding breast cancer and NASH/NAFLD, provides another instance in which IGFBP5 appears to have a highly context dependent mechanism which is in some cases pro-tumorigenic. In the previous section, IGFBP5 and Wnt were determined to be anti-tumorigenic in breast cancer, however, studies in colon cancer report opposing results. Tetrandrine, a natural product, inhibits proliferation and induces apoptosis through inhibition of the Wnt pathway in several cell lines derived from patients with colon cancer. Specifically, tetrandrine downregulates IGFBP5, and exogenous IGFBP5 expression partially rescued the Wnt activity that was lost with tetrandrine treatment (91). However, this work has yet to be expanded beyond an *in vitro* experimental setting, and the directionality of the signaling cascades differ between these studies. In breast cancer, Wnt was thought to be upstream of IGFBP5 to inhibit tumor growth, however in colon cancer Wnt is thought to be downstream of IGFBP5 to promote cell growth. Studies further elucidating the relationship between IGFBP5 and Wnt, and possibly establishing a feedback loop, could help clarify these findings.

The oncogenic activity of IGFBP5 can also be influenced by microRNAs (miRNAs), which are frequently dysregulated in human malignancies. For example, miR-204-5p normally suppresses IGFBP5, however miR-204-5p is downregulated in papillary thyroid carcinoma (PTC), thereby enhancing IGFBP5 expression. Studies demonstrated that IGFBP5 overexpression abrogated the miR-204-5p induced decrease in cell viability and colony formation, and reversed miR-204-5p induced apoptosis, indicating that miR-204-5p could be acting as a tumor suppressor through its downregulation of IGFBP5 (94). In infantile hemangioma, miR-137 was found to regulate IGFBP5 expression, and this signaling axis is under the control of the long-noncoding RNA taurine upregulated gene 1 (TUG1) (104). TUG1 knockdown resulted in increased miR-137, which decreased expression of IGFBP5, and inhibited tumorigenesis *in vivo*, indicating that TUG1 acts in an oncogenic manner potentially through the upregulation of IGFBP5 (104).

Prostate cancer is the second leading cause of cancer-related deaths among men and begins in an androgen dependent state. Following androgen removal therapy, cancer cells may develop androgen independence, at which point the disease becomes fatal. IGFBP5 overexpression increases the progression to androgen independence in subcutaneous tumors in nude mice after castration, and IGFBP5 silencing with antisense oligodeoxynucleotides reduced proliferation in Shionogi tumor cells. IGFBP5 silencing similarly reduced growth of recurring tumors and reduced Elk1 phosphorylation and subsequent MAPK activation (105). Androgen replacement *via* testosterone stimulation of castrated mice secondary to prostate cancer xenografts caused an increase in IGFBP5, indicating IGFBP5 may be regulated by the androgen receptor (AR) in some cases (106).

## 9 Role of IGFBP5 in therapy resistance

### 9.1 Breast cancer

A common mechanism of drug resistance in malignant cells is the acquisition of mutations in the PI3KCA gene resulting in aberrant PI3K/AKT signaling (107), a pathway downstream of the IGF-1R (**Figure 1**). Small molecule PI3K inhibitors have been investigated in clinical trials with limited success (108). A recent study evaluating mechanisms of PI3K inhibition in breast cancer revealed that the histone H3K27me3 demethylase KDM6B acted *via* IGFBP5 to confer resistance to PI3K inhibition and evasion of apoptosis (109). IGFBP5 may also be important in maintaining sensitivity to hormonal therapies such as tamoxifen, which is a selective estrogen receptor modulator (SERM) that, in breast tissue, inhibits binding of estrogen to the estrogen receptor. One study found that IGFBP5 expression is significantly decreased in tamoxifen-resistant breast cancer cells relative to treatment-sensitive cells (110), while another showed that histone demethylase retinoblastoma-binding protein 2 (RBP2) decreases IGFBP5 expression, thereby hyperactivating the IGF signaling axis resulting in tamoxifen resistance (111).

These studies again highlight the context specific nature of IGFBP5 in mediating effects of different targeted therapies. Whereas IGFBP5 reduces effectiveness of PI3K inhibitors, IGFBP5 enhances the effectiveness of tamoxifen in ER+ breast cancers. These effects can be potentiated by stromal cells in the TME such as mesenchymal stem cells and cancer associated fibroblasts (CAFs) that can regulate IGFBP5 levels. For example, MCF-7 breast cancer cells treated with CAF conditioned media had decreased expression of IGFBP5 that was exacerbated by treatment with fulvestrant, another anti-estrogen therapy. Both CAF-mediated downregulation of IGFBP5 and IGFBP5 silencing *via* siRNA resulted in upregulation of B-cell lymphoma 3 protein (Bcl-3), an oncogene associated with unfavorable outcome in patients with ERα/PR-positive tumors subjected to endocrine therapies (81).

Anti-diabetic therapies such as metformin have been employed as an adjuvant therapy in management of breast cancer (112). The recent I-SPY2 clinical trial evaluated the addition of novel agents in combination with paclitaxel chemotherapy and adjuvant metformin and found that found that low gene expression of IGFBP5 in pre-treatment biopsies was associated with better response to ganitumab, a monoclonal antibody against the IGF-1R (113).. Although this regimen had an increase in pathologic complete response in HR-/HER2- breast cancer patients, it did not meet the threshold for continuation to Phase II testing.

### 9.2 Prostate cancer

High-dose external beam radiotherapy is the standard of care for patients with localized prostate cancer and is often used as a combination therapy with androgen deprivation therapy (ADT), however this combination treatment carries considerable risks and the possibility of severe side effects (114). Clinical studies in prostate cancer show that increased expression of IGFBP5 improves the efficacy of radiotherapy for localized prostate cancer, and *in vitro* IGFBP5 overexpression induced G2/M phase cell cycle arrest *via* PI3K/AKT signaling (87). By increasing the efficacy of radiotherapy as a monotherapy, IGFBP5 treatment could present a viable option for prostate cancer patients to subvert the need for ADT.

### 9.3 Ovarian cancer

Ovarian cancer is most frequently diagnosed at an advanced stage and over 75% of these patients will relapse within five years (115), eventually developing resistance to platinum-based chemotherapies. Ovarian cancer cells consistently express the estrogen receptor and studies are exploring the use of hormone therapy as a treatment option for patients who have acquired resistance to front-line chemotherapies (116). A comparison of immunoscores measuring protein expression showed that 17β-estradiol (E2), a natural estrogenic steroid, downregulated IGFBP3 and IGFBP5 while simultaneously upregulated IGFBP4, and this modulation was reversed by tamoxifen (117).

Although IGFBP5 has not been studied in the context of drug resistance in ovarian cancer, a bioinformatic analysis of cisplatin resistant ovarian cancer cell lines identified IGFBP5 as significantly downregulated when compared to the parental cell line (118). Similarly, in esophageal cancer, cisplatin resistant cells have lower IGFBP5 relative to cisplatin sensitive cells, and reintroducing IGFBP5 resulted in a 41% reduction in acquired cisplatin resistance (119). Taken together these studies suggest IGFBP5 may be important for sensitizing cancer cells to platinum-based cytotoxic therapies.

## 10 Discussion

**Figure 7:**
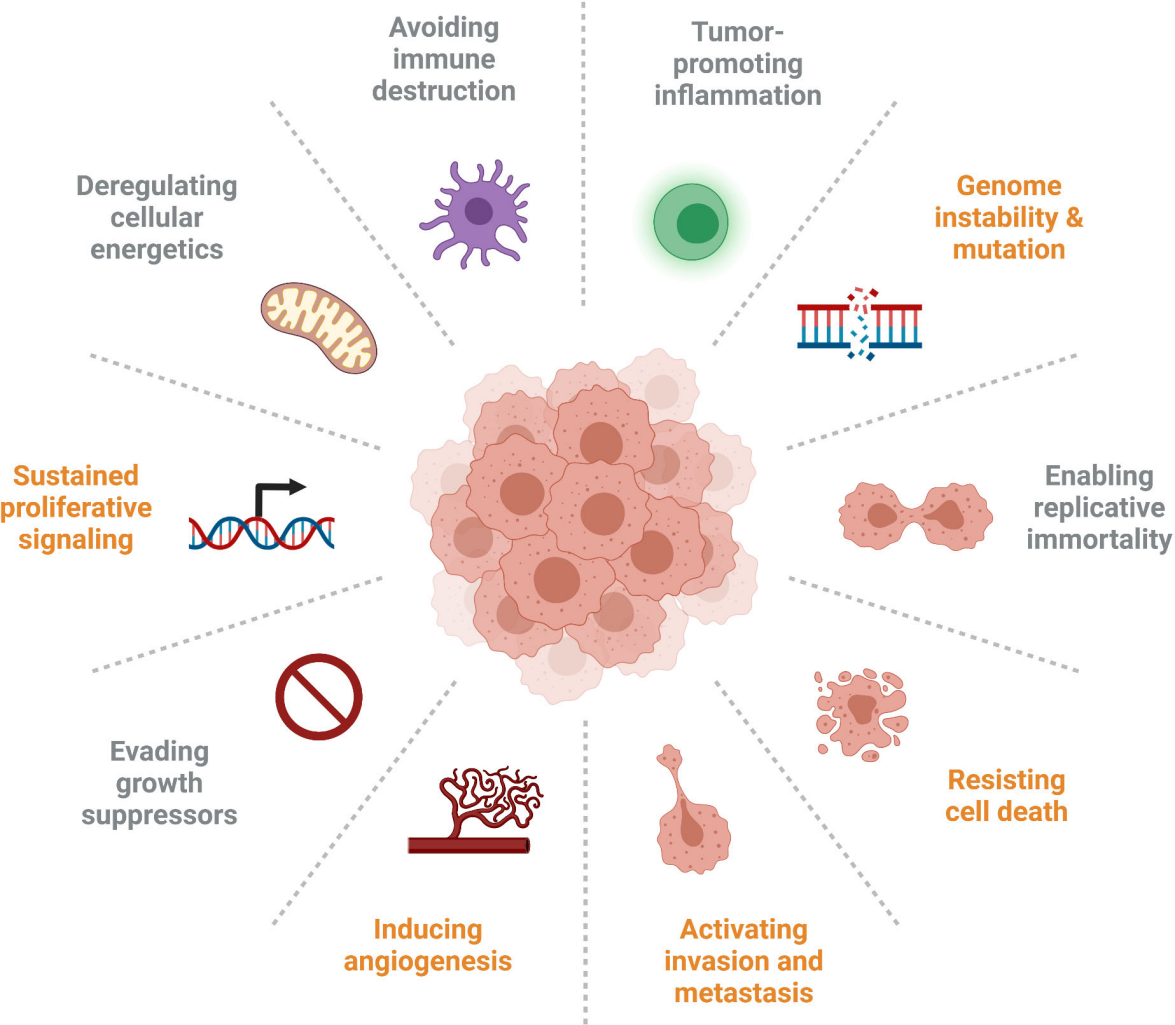
Several of the Hallmarks of Cancer are impacted by aberrant expression of IGFBP5 (identified in orange), which affects cell proliferation and survival, inhibits angiogenesis, enables adhesion of metastatic cells, and is prone to copy number variation in clinical samples.

IGFBP5 has numerous diverse functions that are important in cancer progression, and the signalling pathways modulated by IGFBP5 and their resultant phenotypes are implicated in five out of ten of the updated Hallmarks of Cancer that were expanded upon in 2011 (**Figure 7**) (120). Specifically, the studies explored in this review highlight roles for IGFBP5 in the following cellular defects that enable neoplasia: sustained proliferative signalling, induction of angiogenesis, ability to resist cell death, activation of invasion and metastasis, and genome instability and mutation.

Canonically, IGFBP5 inhibits activation of the IGF signalling axis leading to reduced proliferation and angiogenesis. This mechanism is supportive of an anti-tumorigenic phenotype in ovarian cancer with increased IGFBP5 leading to reduced angiogenesis and growth *via* reduced VEGF activity (74). Overexpression of IGFBP5 also causes breast cancer cells to undergo apoptosis by increasing Bax and decreasing Bcl-2, thus regulating the ability to resist cell death (121). The noncanonical activities of IGFBP5 promote cellular attachment to ECM proteins, a phenotype associated with invasion and metastasis, and ectopic expression of IGFBP5 results in decreased invasion of melanoma and breast cancer cells. The prevalence of CNVs in numerous cancers indicates some genome instability. The involvement of IGFBP5 in all these various pathways to neoplasia illustrate the importance of this protein, and indeed this family of proteins, in regulating the development and progression of cancer.

Although IGFBP5 has several established mechanisms in cancer cells, the investigation of IGFBP5 in cancer progression should be expanded to include more components of the TME. Stromal cell populations in the tumor, such as fibroblasts, are also responsible for IGFBP5 secretion, and study of fibroblast-induced changes in IGFBP5 expression in the TME could be informative in the context of epithelial-to-mesenchymal transition (EMT). It is worth noting that the models used to date are limited by their reliance on permanent changes in IGFBP5 gene expression, either *via* stable overexpression or gene knockout. Permanent experimental manipulation of IGFBP5 levels do not permit an assessment of the temporal modulation that may occur in response to stromal cells or epigenetic modifiers. This highlights the need to design experiments that consider spatial and temporal factors which influence cancer progression and to include assessment of stromal compartments.

Given that IGFBP5 is pro-fibrotic and can be anti- or pro-tumorigenic depending on the cancer subtype, the differentiation status of the cell, TME, and circumstances of activation, studies of this protein should also consider the context in which the protein is functioning and whether it is acting in an IGF dependent or independent manner. IGFBP5 promotes the production of secreted matrix proteins such as collagens and glycosaminoglycans and can be associated with the matrix indicating that it could assist in the “docking” of cancer cells to distant sites, which is an example of one of the proteins IGF-independent mechanisms. Some studies have suggested that IGFBP5 could bind to substrates including vitronectin, thrombospondin and osteopontin while still bound by IGF, creating a reservoir of available IGF in the TME (122), but other studies have demonstrated that when IGFBP5 binds to ECM components, it greatly reduces its affinity for IGF (34). More work is needed to demonstrate this is a valid mechanism in cancer progression, notably that these complexes exist in the TME *in vivo*. This could be of particular interest in cancers which initiate in or metastasize to ECM-rich tissues, such as ovarian and pancreatic cancers which metastasize into the abdominal cavity with a wide variety of substrates available for subsequent cancer cell adhesion.

MMPs additionally must be considered as an important regulator of the fibrotic stroma in the TME which are involved in proteolytic cleavage of IGFBP5. The domains of IGFBP5 have differential activity in cancer as previously discussed, and MMPs and other proteins that cleave IGFBP5 could influence the impact of this protein has in cancer by increasing the prevalence of protein fragments. Further investigation characterizing the role of proteolytic enzymes in regulating the aberrant activation or degradation of IGFBP5 in cancer is needed to better understand the role of the individual subdomains of IGFBP5, as is proteomic analysis of patient derived samples that quantify the relative abundance of various IGFBP5 fragments relative to the intact protein.

IGFBP5 can also be regulated by epigenetic mechanisms that are responsive to extrinsic factors. For example, downregulation of DNA methyltransferase 3A (DNMT3A) results in increased IGFBP5 in patients with preeclampsia due to hypomethylation of the IGFBP5 promoter (123). Another methyltransferase, enhancer of zeste homolog 2 (EZH2), also represses IGFBP5 expression, and after conditional knockout IGFBP5 was elevated in uterine epithelium relative to wild type mice (124). Thus, it is conceivable that IGFBP5 expression is dynamic and potentially acting as a switch that can either aid or inhibit tumor progression, depending on factors such as promoter methylation.

Due to the similarities between IGFBP5 and the other IGFBPs, as well as the IGF binding CCN family, there may be compensatory mechanisms that have not yet been elucidated that contribute to the context-specific effects of IGFBP5 in cancer progression. The data indicating that increased IGFBP5 is correlated with decreased overall survival in ovarian (62), breast (69) and other cancers (125) contrasts with the many *in vitro* and *in vivo* studies suggesting a tumor-suppressor function. Understanding IGFBP5 activity in different contexts including cell type, differentiation status, microenvironment, or in conjunction with other pro-tumorigenic pathways will provide mechanistic insight into the role of IGFBP5 in cancer progression. Altogether the findings reviewed here indicate a need for a more comprehensive evaluation of IGFBP5 to understand its association with poor outcomes in cancer patients and identify new potential markers and/or therapeutic avenues.

## Author contributions

JW and CH designed the work. JW wrote the manuscript. JW, MR and IU prepared the figures and tables. JW, MR and CH drafted and revised the manuscript. All authors contributed to the article and approved the submitted version.

## Funding

This work was funded by the National Institute on Minority Health and Health Disparities at the NIH under award number U54MD012397.

## Acknowledgments

JW would like to acknowledge the support from the ARCS Foundation and the Rees-Stealy Research Foundation. The authors would like to acknowledge that **Figures 1**-**4** and **7** were created with BioRender.com.

## Conflict of interest

The authors declare that the research was conducted in the absence of any commercial or financial relationships that could be construed as a potential conflict of interest.

## Publisher’s note

All claims expressed in this article are solely those of the authors and do not necessarily represent those of their affiliated organizations, or those of the publisher, the editors and the reviewers. Any product that may be evaluated in this article, or claim that may be made by its manufacturer, is not guaranteed or endorsed by the publisher.
